# Alignment of Goals for Personal Interpretation Among Staff Groups in a Park Agency

**DOI:** 10.1177/10925872231158092

**Published:** 2023-05-24

**Authors:** Glen T. Hvenegaard, Elizabeth A. Halpenny, Clara-Jane Blye

**Affiliations:** 1 University of Alberta, Camrose, AB, Canada; 2 University of Alberta, Edmonton, AB, Canada

**Keywords:** interpretation, parks, goals, outcomes, goal alignment, staff, priorities, Alberta Parks

## Abstract

Goal alignment among staff members helps an agency perform well. This study examines goal alignment for park staff regarding their priorities for interpretation and perceptions of what helps and hinders achievement of those goals. We surveyed 86 staff from Alberta Parks, who rated potential goals for personal interpretation and expressed views on the catalysts and constraints that affected the achievement of those goals. There was alignment in interpretation goals among staff groups (i.e., planners/managers, interpretive supervisors, and frontline interpreters). Staff thought that all goals were important, but ranked the goals of positive memories, enjoyment, and connections to place higher than the goals of behavior change, positive attitudes, and learning. Factors supporting success were supportive supervisors, hiring and retaining excellent staff, and training, whereas factors hindering success were resource deficits, bureaucracy, and lack of common goals. To promote goal alignment, agencies can improve communication, planning, staff engagement, training, and research.

## Introduction

Personal interpretation is widely believed to increase enjoyment for visitors, as well as promote stewardship behaviors and increase knowledge of natural and cultural resources (Powell et al., 2009; Stern & Powell, 2013). However, with park agencies experiencing reduced budgets and resources, it is important to prioritize outcomes and efficiently pursue them. For most park agencies, the mandate for providing interpretive activities comes from their enabling legislation (Wade, 2010). However, even with clearly identified guiding policies, policy-makers, planners, managers, and field staff perceive interpretive goals in different and potentially incompatible ways. Such varying perceptions may result in misalignment between management and interpreters, which may, in turn, influence outcomes and experiences for park visitors (Benton, 2011).

Even when goals are aligned, many catalysts and constraints affect the achievement of these goals. Agency personnel’s perceptions of these factors are important sources of information that can be used to accelerate achievement of desired interpretation program outcomes. This paper advances efficient and effective agency attainment of desired interpretation outcomes through a case study of a Canadian protected area agency; it reports on the (a) alignment of personnel interpretive goals and priorities and (b) staff perspectives of what supports or inhibits attainment of interpretation program outcomes. Lessons that arise from this case can be employed in other park agencies that wish to reconcile and prioritize interpretation program goals and outcomes.

### Goal Alignment in Organizations

Organizational goals (desired outcomes) that can guide action and evaluate performance, are found in mission statements, strategic plans, corporate strategies, and employee priorities (Kotlar et al., 2018; Torland et al., 2015). However, the goals stated by an organization can be different from the goals driving employees (Gowan et al., 2001; Kotlar et al., 2018) and can vary over time (Bode et al., 2011; Dayer et al., 2016; Kotlar et al., 2018). Goal alignment entails “linking individual goal outcomes with organizational goal outcomes” (Ayers, 2015, p. 170). The alignment of employees’ goals with an organization’s goals improves the organization’s performance (Ayers, 2015). A major obstacle to strategic implementation in an organization is a lack of consensus between top-level management and other staff in an organization (Rapert et al., 2002). The lack of goals, setting of ambiguous goals, and poorly aligned goals can impede performance and staff satisfaction (Chun & Rainey, 2006; Davies et al., 2004).

Fisheries and medical research has shown that the lack of understanding of goals and objectives within the organization can lead to interaction conflicts among staff (Barber & Taylor, 1990) and that goal misalignment between education and delivery can result in over-spending and a lack of innovation (Sklar et al., 2018). In federal agencies, goal alignment can help employees’ day-to-day performance, promote the achievement of strategic goals, and support links between desired goals (Ayers, 2015).

Some organizational characteristics can affect goal alignment. Employees at different hierarchical levels have varying perceptions of goals. For instance, regarding a business’s ethics, executives provided the most positive assessments, low-level employees provided the least positive assessments, and mid-level managers provided moderate assessments (Ardichvili et al., 2012). However, experience with a variety of positions in an agency does not lead to greater alignment with an organization’s objectives (Boswell, 2006). Several employee characteristics can also help predict goal alignment. In a study of public and non-profit organizations in the NE United States, goal ambiguity decreased as age increased (Sun et al., 2014). Similarly, more experience at a health care agency was related to more consistency between the employee and the agency’s strategic objectives (Boswell, 2006). Last, in a forestry organization, staff training increased goal alignment (Ries, 2020). Unlike other government departments or organizations, there appears to be little empirical research on park agencies’ goal alignment, and especially as it relates to the delivery of personal interpretation. This is important as interpretation has been shown to be more effective when goals are in place and planned for (Stern & Powell, 2013, 2021).

### Goal Alignment and Interpretation

Whether the focus is on personal or non-personal interpretation (Hvenegaard & Shultis, 2016), the most researched goals of interpretation for visitors are learning, attitude change, satisfaction or enjoyment, engagement with interpretation, behavioral intentions, and behavior (Stern & Powell, 2021). Other common goals include connections to place and positive memories (Blye et al., 2022).

Studies on goal alignment in interpretation have produced mixed results. Stern and Powell (2011) found agreement between park superintendents and interpretive staff about the goals of interpretation in U.S. National Park Service units (the top three goals were revelation/inspiration, stewardship/support, and visitor enjoyment). However, the perceptions of frontline interpreters may differ from those of other groups of employees, or those of the agency (Machnik et al., 2006). In Alberta, there were differences between park policy goals and the goals of those who plan and deliver interpretive programs (Blye et al., 2022). Moreover, goals for interpretation may vary by career stage among park staff. For instance, the goals of entry-level environmental education staff focused on information, understanding, policy awareness, enjoyment, and appreciation, while goals of staff at higher levels focused on environmental awareness and behavior change (Knapp et al., 1997). Once environmental educators are hired, employers can encourage goal alignment by communicating organizational values and norms, promoting positive relationships among staff, identifying clear career paths, and stressing the role individuals can play in organizational goals (Pratson et al., 2021). It is valuable to address goal alignment in park interpretation in order to improve individual performance, enhance employee contributions, and improve organizational performance.

### Factors Helping and Hindering Goal Achievement

Several organizational and personal factors can support. or constrain the achievement of interpretive goals. Herzberg’s (1966) motivation-hygiene theory suggests that hygiene factors (i.e., organizational policies, work conditions, and relationships among employees) help organizations to operate (and employees to perform), but do not result in long-term motivations to achieve goals. Additionally, motivation factors highlighted in Self Determination Theory (i.e., sense of achievement, advancement, recognition, and meaning in the work; Deci & Ryan, 1985) may be more relevant in making progress toward goals.

The utility of these organization and personal theories for understanding advancement of organizational goals and effectiveness are supported by empirical research. A study of government employees and organizational commitment provides confirmation of the importance of personal factors; earlier career employees appeared motivated by supervisory support, whereas employees in later career stages were driven by opportunities for professional advancement (Aryee et al., 1994). Clement et al.’s (2016) analysis of the efficacy of cross-border protected areas highlighted the impact of personal factors such as staff’s ability to navigate political influence, but also organization factors such as restrictions on staff authority and discretion. Another group of organizational factors that affects effectiveness and goal prioritization relates to resourcing. Research on southeast Asian protected areas suggests management effectiveness and specifically biodiversity conservation outcomes are strongly associated with financial and human resourcing as well as with access to equipment (Graham et al., 2021). A recent review of 41 protected areas in Queensland, Australia observed that budget shortfalls undermine biodiversity conservation, and when managers are forced to prioritize actions, they appear to privilege visitor experiences over biodiversity management (Craigie & Pressey, 2022).

Studies that document staff insights about factors that affect the efficacy (goal attainment) of environmental education and interpretation programs within protected areas are scarce. In a review of factors that motivated environmental educators, Pratson et al. (2021) observed that many organizational practices influenced feelings of autonomy (e.g., freedom), competence (e.g., training), relatedness (e.g., meetings), meaningfulness (e.g., encouragement), and novelty (e.g., variety of tasks). Environmental educators early in their career were more motivated by opportunities to grow professionally, whereas educators later in their career were more motivated by variety in their tasks (Pratson et al., 2021). In a study of two parks in Columbia, Zorrilla-Pujana and Rossi (2014) noted the importance of aligning environmental education efforts with biodiversity conservation and management objectives. Given the diversity of factors, both organizational and personal, that can influence protected areas’ goal attainment, and the varied observations documented across operating contexts, further examination of park employee perspectives about what constrains or promotes interpretation program success seems warranted. This is especially true given the paucity of studies that attempt to draw insights from protected area staff.

### Research Aims and Importance

The aims of this study were twofold. First, what do interpreters and park managers believe to be the most important goals of interpretation in Alberta Parks, and do staff in various positions see these differently or the same? Second, what do interpreters and park managers believe are the key catalysts and constraints to achieving these goals, and do staff in various positions see these differently or the same? We chose to examine a single park agency, Alberta Parks, which provides cultural and natural heritage interpretation. We focused on personal interpretation, which links to our larger research program focused on visitor perceptions of personal interpretation.

This study is important because the results can help agencies improve communications, coordination, and provide support to employees. In addition, few studies have shown how the importance of interpretation goals vary among park employees. These results can help agencies diagnose underperformance by understanding where goal misalignment is occurring (Ayers, 2015; Voss et al., 2006). If the importance of goals and organizational support (or lack thereof) vary among employees, managers can encourage goal alignment in order to increase agency performance.

## Methods

This study employed a convergent mixed methods design (Creswell & Plano Clark, 2018) which provides insight into a problem or question by utilizing the strength of both quantitative and qualitative methods (Creswell & Creswell, 2017). As a result, both qualitative and quantitative data are collected in a single phase, analyzed separately, and compared (Creswell & Plano Clark, 2018).

### Study Site

Alberta Parks, the agency responsible for 476 protected areas across the province, has a mandate to “inspire people to discover, value, protect, and enjoy the natural world and the benefits it provides for current and future generations” (Government of Alberta, 2009, p. 3). Interpretation programs offer “an opportunity to learn about, appreciate, and care for natural and cultural heritage” (Government of Alberta, 2009, p. 17). Delivering and administering interpretation and environmental education normally involves 17 full-time and approximately 45 seasonal frontline staff (Alberta Parks, 2016). Before the pandemic, Alberta Parks received about 9 million visits each year, including 450,000 associated with education and interpretive programs (Alberta Parks, 2016).

### Sampling and Data Collection

Our research subjects came from two sampling groups. **Sampling group 1** consisted of full-time employees of Alberta Parks who were involved in making decisions about personal interpretation. We identified candidates from the staff directory of the government Department of Environment and Parks (Government of Alberta, 2021). Two senior Alberta Parks staff involved in interpretation reviewed our list to confirm that candidates were appropriate, and to suggest additional names. Our staff categories were planners and managers (employees engaged in province-wide planning exercises or making park-related decisions), interpretive supervisors and coordinators (employees supervising or coordinating frontline interpreters), and frontline interpreters (employees delivering interpretive programs to park visitors). This resulted in a target list of 54 potential respondents. For participants within sampling group 1, we conducted structured interviews, via phone or an online communication system from February to July 2021. Structured interviews can act similar to a survey in oral form but provide the opportunity for more in-depth answers and allow for both closed and open-ended questions (Fontana & Frey, 2005). We conducted 38 structured interviews (60.5% were planners and managers and 39.5% interpretive supervisors and coordinators), with a response rate of 70.3%. The average interview lasted 43 min (range = 25–78).

**Sampling group 2** consisted of full-and part-time interpretation employees who were required to attend a province-wide staff training event in May 2018 (just prior to the season of delivering personal interpretation). At this event, we asked 46 staff to complete a self-administered questionnaire (response rate of 100%) (41 were frontline interpreters and 5 were interpretive coordinators or supervisors). The questionnaire was a text version of the structured interviews, but excluded questions not relevant to frontline interpreters. Only data generated by the questions asked to both sampling groups were included in this paper.

With an open-ended question, we asked all respondents to describe what they felt were the most important goals of personal interpretation for visitors. With a closed-ended question, we then asked respondents to rate the importance (1 = not important to 7 = extremely important, with no internal anchors) of six goals (enjoyment of the experience, learning about the topic, achieving a positive attitude toward the topic, behavior change toward the topic, increased connections to the place, and making positive memories). The first four items came from past research (Benton, 2009; Hvenegaard, 2017; Skibins et al., 2012) and the last two came from interviews with senior park interpreters with Alberta Parks (Blye et al., 2022; Cook et al., 2021). With another closed-ended question, respondents also indicated their single most important goal from the same list of six goals. We also asked respondents to indicate, via open-ended questions, what factors most helped or hindered the achievement of personal interpretation outcomes.

Finally, we asked about each respondent’s birth year, gender, education level, discipline of training, current staff position, impact of current position on personal interpretation, years in this role, years supervising interpreters (if any), and location of current position (which park or headquarters).

### Data Analysis

We analyzed quantitative data using SPSS 28.0 (IBM Corporation, Armonk, NY). We calculated respondent age (current year minus birth year), categorized educational levels, categorized discipline of education, placed current positions into three categories, categorized experience levels, and noted the location of work assignment (parks vs. provincial headquarters). We used independent samples t-tests, one-way ANOVA tests, and chi-square tests, to explore relationships, based on a *p*-value threshold of .05.

From each respondent, we also received qualitative data about the importance of interpretive goals and factors that helped or hindered success in delivering interpretive programs. The research team transcribed all responses, read them in their entirety, and coded for themes using NVivo 12. Data analysis followed Mayring’s (2004) content analysis, characterized by systematic examination of communicative material, with the results derived inductively from the text. If a respondent indicated two goals or factors simultaneously as “most important,” then both were counted. Thus, some totals for interpretation goals and decision-making resources were higher than the number of participants interviewed.

## Results

Combining both sampling groups (*n* = 86), 55.8% were females, 41.9% were males, and 2.4% indicated other categories or did not provide an answer. Respondents’ mean age was 36 years (range = 20–63). In terms of education, 16.3% had a college diploma, 7.0% had some university, 57.0% held a bachelor’s degree, 11.6% held a master’s degree, and 8.1% held a doctorate. The most common educational disciplines were agriculture or natural resources conservation (37.2%), visual/performing arts (12.8%), education (12.8%), physical and life sciences (11.6%), humanities (8.1%), and health/parks/recreation (7.0%).

Most respondents (60.5%) had other training in personal interpretation (e.g., formal courses, conferences, or workshops). The respondents represented all provincial park administrative regions. Most respondents (65.1%) worked at specific parks, while 34.9% worked at the provincial headquarters. By position, our sample included planners and managers (27.9%), interpretive supervisors and coordinators (24.4%), and frontline interpreters (47.7%). Respondents had worked a mean of 3.6 years in these positions (range = 1–25). Planners/managers (5.3 years) and coordinators/supervisors (6.2 years) had more experience than frontline interpreters did (1.2 years; F = 12.7, p < .001, d = 0.22). Only 37.2% of the sample had supervised frontline interpretive staff at some point in their careers, but this rate differed among planners/managers (50.0%), coordinators/supervisors (85.7%), and frontline interpreters (4.9%; X^2^ = 41.2, df = 2, p < .001). Of those who had supervised frontline interpreters, the mean number of years of supervising interpreters was 10.3 (range = 1–30) with no differences by staff group observed.

### Goals of Interpretation

Respondents indicated the importance of interpretive goals in three ways. First, using open-ended questions, we asked each participant to describe their own goals for interpretation. Asking participants to reflect on any possible goals allowed for the emergence of goals missed in previous studies and for a better understanding of what each goal meant to participants. Table 1 describes the five main goal themes. Respondents emphasized stewardship, connections to place, memorable experiences, increased desire for learning, and behavior change. Some respondents identified more than one goal.

**Table 1.** Themes of the Most Important Visitor Goals of Personal Interpretation as Identified by Staff (Based on an Open-Ended Question).

Goal	Theme description	Sample quotation
Stewardship	Interpretation should foster a deeper understanding and appreciation of parks and protected areas. Programs and content should highlight key environmental issues and provide meaningful direction for addressing these issues. Visitors should be inspired to act, and they should share that passion with others.	“Increase appreciation for the value that parks provide, understand how important the parks system is to economic, social, and environmental values. Make parks relevant so people want to support them”—Participant 28 (manager director) “End with a call to action, some people might take it back home and help protect nature”—Participant 81 (frontline interpreter)
Connections to place	Interpretation should foster connections to parks and to nature more broadly. Interpretation should build content and programs that connect visitors to the local site and that facilitates behavior change and a sense of caring for nature.	“Facilitating a deep connection to the site (park) and a deeper connection to the stories of that landscape. Help visitors develop a personal relationship with nature”—Participant 33 (manager)
Memorable experiences	Parks should provide programs that are memorable, inclusive, positive, and ignite passion. Visitors should remember their programs and experiences long after leaving the park. Respondents described the desire for multi- generational attendance and noted the desire for visitors to recall childhood interpretive experiences.	“Transformative experiences, we are doing interpretation for change and facilitating memories—remembering the experiences in our parks”—Participant 36 (manager)
Increased desire for learning	Interpreters should provide free choice learning opportunities that spark interests and creativity. Participants felt that interpretation should create a desire to learn more.	“Inspiring people to explore, giving people the knowledge so that they can go deeper on their understanding of nature”—Participant 13 (coordinator or supervisor) “We want people to walk away from the program and they should have learned something they didn’t know and added enrichment to their own lives”—Participant 16 (planner)
Behavior change	Effective communication can influence values, attitudes, and visitor behaviors, both on and off sites. Many respondents wanted to use interpretation as a tool to increase pro-environmental behaviors.	“Interpretation is powerful when we see behavior change. When we finally have people realize that they have an impact and have a personal responsibility to change their behaviors”—Participant 24 (coordinator or supervisor)

Broken down by staff category, staff spoke overwhelmingly of the importance of promoting stewardship, connections to place, and memorable experiences for park visitors. Learning and behavior change appeared to be less important for most staff categories. Interestingly, when asked what their perceived main goal of interpretation is, behavior change appeared not relevant to frontline staff.

Second, using a closed-ended Likert-scale question, we asked respondents to rate the importance six pre-determined interpretive goals. Each goal received a mean rating of 5.6 or higher out of 7 (Table 2). Positive memories, enjoyment, and connections to place received the highest importance scores, while behavior change, learning, and positive attitudes received lower scores. Responses about the importance of interpretation goals varied only for the positive memories goal, which was rated higher by coordinators and supervisors than by planners and managers (F = 3.10, df = 85, p = .05; Table 2).

**Table 2.** Ratings of Importance (1–7) for Six Pre-Set Interpretation Goals by Staff Category.

Goal	Mean (*SD*)	Planners and managers *n* = 24	Interpretive coordinators and supervisors *n* = 21	Frontline interpreters *n* = 41
Positive memories	6.7 (0.7)	6.4^a^	7.0^b^	6.6^a,b^
Enjoyment	6.7 (0.6)	6.5	6.5	6.8
Connections to place	6.4 (0.9)	6.3	6.5	6.5
Behavior change	5.8 (1.2)	5.8	6.2	5.6
Positive attitude	5.7 (1.1)	5.6	5.7	5.8
Learning	5.6 (1.1)	5.5	5.3	5.9

*Note*. Responses to the question “Rate the importance of the following six interpretation goals. . . .”.

a,b Indicate means are significantly different.

Third, we asked which of the six pre-determined interpretation goals was most important. Most respondents (39.5%) rated connections to place as the most important, followed by positive memories (23.3%), enjoyment (18.6%), behavior change (11.6%), learning (4.7%), and positive attitude (2.3%).

### Factors That Helped or Hindered Achieving Interpretation Goals

To understand how park staff achieve goals and develop interpretive programing, we asked respondents to reflect on what they felt helped or hindered success. Responses provided descriptions of organizational factors identified through lived experiences of delivering, planning, or managing personal interpretation. Tables 3 and 4 provide insight into these categories and offer examples of how each category was described by participants.

**Table 3.** Factors that Helped Achieve Interpretation Goals (Based on an Open-Ended Question of Alberta Parks Staff).

Category	Category description	Sample quotation
Support from supervisors	Respondents from all staff categories mentioned the support and encouragement provided from supervisors and management. As a result, staff felt valued and empowered in their roles and felt validated in their fields of interpretation and environmental communication.	“Supportive managers, to try some new things with my programs has had a big impact. All the way up to the director level are supportive of our (interpretive) programs in general and really see the value in it, they might not understand it fully but they do seem to know there is value to it.”—Participant 33 (manager)
Excellent interpretive staff	Respondents described successful delivery of interpretation as highly correlated to the “amazing” team. The ability to re-hire returning staff and recruit and hire new staff, all of whom have great knowledge, experience, and passion, were cited as core elements for success.	“Recruiting good staff, we have a good process for that. Hire the best people you can get and try to stay really current. It is not just about getting the best people in Alberta but all over Canada.”—Participant 32 (manager) “Collaboration and support from the entire interpretive staff team”—Participant 66 (frontline interpreter)
Training	Staff cited both the ongoing and seasonal training session as a significant contributor to success. Seasonal and permanent staff from the agency participate in an annual 3–4 days interpretation training retreat (*in situ*). This training provides education, supports collaboration, and builds a supportive team environment,	“The most extraordinary training I have ever received!”—Participant 74 (frontline interpreter) “Alberta Parks respects and puts a lot of effort into their (seasonal) staff, we have lots of support for training and education”—Participant 13 (supervisor or coordinator)

**Table 4.** Factors that Hindered the Achievement of Goals for Personal Interpretation (Based on an Open-Ended Question of Alberta Parks Staff).

Category	Category description	Sample quotation
Resource deficits	All respondents (at all levels) cited resources deficits (financial, time, staff, and supplies). Budget cuts affect the ability to hire, retain, and support staff, thereby requiring the remaining staff to do more with less. Respondents mentioned other areas such as program supplies, time to develop new programs and interpretive content, and training. Lack of resources contributes to feeling undervalued and needing to defend interpretive programing within parks.	“People see interpretation as nice to have but not an essential service, we are constantly redefining who are and proving our relevance and why we are so important” Participant 2 (manager)
Bureaucratic barriers	Respondents mentioned that hierarchical systems within government are rigid and slow moving. Approvals and policy direction require “head office” review and seem to lack regional consideration or engagement. The agency’s rigidity made many participants feel that innovation was and is being stifled, thereby hindering the success of interpretation and the ability of staff to achieve desired goals. The political shifts and cycles contribute to inconsistency and upheaval in staffing requirements.	“Overall, we are hindered by not evolving, although it seems some feel it is easier to keep doing what we are doing, rather than critically evaluating what we are doing and what we need to be doing in order to be effective”—Participant 12 (planner) “The biggest hindrance is bureaucracy, the levels of support to go through for even a single small change. Yet the requirements of being empowered as a staff member contradicts getting every word you say signed off on”—Participant 86 (frontline interpreter)
Lack of common goals	Across the agency, respondents cited a lack of strategic planning and communication of goals. Without clear goals, plans, and strategic direction staff are unable to execute their own goals and feel confused or unsupported in any long-term planning.	“No consistency, everyone does their own thing, there is no curriculum, no guiding topics, cultural or geographical. There is nowhere to go and say here are the topics that we all follow. No set goals and outcomes so then we all just develop our own.”—Participant 16 (supervisor or coordinator)

Table 5 highlights the importance that frontline interpreters place on having supportive supervisors and training opportunities. Managers and supervisors of interpreters felt a large source of their success was due to their ability to hire and retain excellent staff; however, this same staff category noted how challenging budget cuts have become and specifically noted a reduction in staff and challenges of retaining excellent seasonal staff. Bureaucracy was most often noted by planners/managers and supervisors of interpreters alike as a hindrance, however based on percentages this problem was more important for supervisors of interpreters than any other staff category. Finally, and most importantly, planners/managers and supervisors of interpreters were most concerned with the lack of strategic direction within the organization and the need for clearer communication about goals. However, it is interesting to note that this issue does appear to be influencing frontline interpreters (or perhaps they are simply not aware of the issues).

**Table 5.** The Number of Mentions for Factors That Helped or Hindered Interpretive Goals Achievement by Staff Category.

Percent of individuals within each theme per staff category			
Category	Planners and managers	Interpretive coordinators or supervisors	Frontline interpreters
Helped achieve goals			
Supportive supervisors	22%	25%	25%
The ability to hire and retain excellent interpretive staff	35%	50%	9%
Training	4%	10%	22%
Hindered achieving goals			
Resources deficits (financial, time, human, program supplies, etc.)	52%	50%	32%
Bureaucracy and obstruction of innovation	31%	35%	17%
Lack of common goals and strategic direction	57%	85%	0%

*Note*. Responses derived from an open-ended question that asked “Describe the most important goals of personal interpretation for visitors.” Percentages are greater than 100% due to participants identifying more than one main goal.

## Discussion

Goal alignment is associated with greater productivity, efficient and effective use of resources, higher staff morale, and more satisfied stakeholders (Ayers, 2015; Barber & Taylor, 1990; Chun & Rainey, 2006; Davies et al., 2004). Park staff perceptions related to agency goals and the factors that constrain and facilitate their achievement are rarely documented. This lack of research is especially apparent for interpretation and environmental education service delivery. This study sought to determine, in the context of goal alignment, the importance of various park interpretation goals among park staff and the factors that helped and hindered achieving these goals among park staff.

### Interpretation Goals

Based on open-ended questions, respondents spoke about the importance of promoting stewardship, connections to place, and memorable experiences, learning, and behavior change for park visitors. However, when asked on a Likert-type scale to rank the importance of a given set of goals, the goals of positive memories, enjoyment, and connections to place ranked higher than behavior change, attitude change, and learning.

In prior research, enjoyment was one of the top three goals highlighted by US National Park Service superintendents and interpretive supervisors (Stern & Powell, 2011). In the tourism field, the goal of offering experiences that produce positive memories has received renewed research attention, with researchers focusing on affect, expectations, consequentiality, and recollection as key dimensions of memorable tourism experiences (Tung & Ritchie, 2011). Positive memories have been highlighted as an important catalyst for retaining information (Ballantyne et al., 2011); positive reflections help trigger recollection of achievements, knowledge, and skills associated with the positive memories. The memories can lead to pro-environmental action (Liddicoat & Krasny, 2014), destination loyalty (Loureiro, 2014), behavior change (Organ et al., 2015), and place attachment (Scannell & Gifford, 2017).

Regarding the connections to place goal, place-based education is a prevalent topic in environmental education that focuses on school programs and local residents (Dean, 2021; Gruenewald & Smith, 2014). Interpretive staff of Alberta Parks may be influenced by place-based education, as most of their frontline interpreters engage with visitors in on-site interpretation programs and on-site school curriculum delivery. Staff may also be aware of interpreters’ increased use of protected areas’ attributes to accelerate visitors’ awareness of urgent environmental issues such as climate change, demonstrated by disappearing glaciers, pest infestations, and more intense wildfire (Davis, 2014; Gislason et al., 2021; He & Hinch, 2021).

Interpretation programs frequently succeed in increasing learning among attendees (Skibins et al., 2012). Interestingly, learning was less important for the 41 frontline interpreters who responded to our survey; rather stewardship and enjoyment were described as their most important interpretation goals. Enjoyment was the most important goal from the pre-determined list (Table 2) but equally important was the emerging theme of stewardship from the open-ended question regarding goals (Table 6). There is a chance to reflect on language and values here; perhaps frontline interpreters did not feel that learning (or other pre-set goals) truly represent what they are trying to achieve through their programs. It is also relevant to highlight the abundant research suggesting that learning and education are not sufficient on their own to influence behavior change (He et al., 2022; Kollmuss & Agyeman, 2002; Lawhon et al., 2013; Ren & Folta, 2016). Interpretive staff may be well aware that learning alone may not achieve desired behavior changes and therefore a focus on stewardship more broadly allows individuals to feel that goals are more attainable and achievable. In addition, Blye et al. (2022) found that priorities of frontline interpreters for enjoyment and learning declined after a season of work. This decline could be because they sought to focus on longer-term impacts or perhaps they experienced a lack of achievement in these goals.

**Table 6.** Most Important Interpretive Goals as Identified by Staff Compared by Staff Category.

	Percentage of staff category within each theme		
Theme	Planners and managers	Interpretive coordinators and supervisors	Frontline interpreters
Stewardship	57%	55%	48%
Connections to place	57%	35%	29%
Memorable experiences	30%	35%	31%
Increased desire for learning	22%	35%	19%
Behavior change	26%	25%	0%

*Note*. Responses derived from an open-ended question that asked “Describe the most important goals of personal interpretation for visitors.” Percentages are greater than 100% due to participants identifying more than one main goal.

Even though attitude change and behavior change are achieved less frequently than satisfaction and learning (Skibins et al., 2012), Alberta Parks staff may not see tangible evidence of these goals. Thus, parks staff may rank attitude and behavior change lower in priority. Perhaps staff want to achieve easy and immediate measurable outcomes for themselves or for their agency. To inform agency reporting requirements and funding decisions, it is much simpler to track satisfaction levels and knowledge change arising from an interpretive program than it is to track long-term behavior change. Given that Stern and Powell (2013) found that prioritizing the goal of knowledge gain in interpretive programs was associated with lower levels of visitor experience and appreciation, perhaps it is appropriate that staff rated learning low as an interpretive goal.

### Goal Alignment Among Employee Groups

Generally, there was substantial goal alignment among park staff. In other studies, Stern and Powell (2011) found goal alignment among park staff, whereas Knapp et al. (1997) found some misalignment. Even though our respondents rated and discussed behavior change, positive attitudes, and learning slightly lower than the other three goals, respondents still rated all of these goals higher than 5.5 out of 7. The only statistical difference for goals among employee groups was that coordinators and supervisors rated positive memories higher than planners and managers did. Studies of other resource management agencies have also indicated varying opinions by employee groups on agency processes and organizational strategies, particularly due to competing accountabilities felt by those employee groups (Stern et al., 2010). With responsibility for managing frontline interpreters, coordinators and supervisors may focus on enjoyment as a goal that is achievable in the short term. As for positive memories, it is possible that coordinators and supervisors are inclined to focus on longer-term outcomes that will influence future park attendance at interpretation programs or visiting parks.

### Factors Helping and Hindering the Achievement of Interpretation Goals

The most important factors that helped park staff achieve interpretation goals were supportive supervisors, skilled team members, and training opportunities. In a separate Alberta-based study, frontline interpreters felt that supportive supervisors, fellow interpreters, and past experiences were the most important resources that helped them make decisions in delivering interpretation (Blye et al., 2022). These resources had the benefits of social interactions, immediacy, opportunity for feedback, advanced knowledge from more experienced staff members, and authority from supervisors.

Practitioners and researchers have long known about the importance of training to develop various competencies related to audience experiences, finding and assessing knowledge, using appropriate techniques, collaboration, planning and evaluation, and continuing professional development (Powell et al., 2017). In particular, interpreters need training to increase research literacy, engage new audiences, and effectively use technologies (Powell et al., 2017). Perceptions about training needs vary slightly by age and employment level (Powell et al., 2017). In our study, frontline employees identified training as the most important factor in support of achieving interpretative goals, while coordinators and supervisors prioritized the retention and hiring of excellent interpretive staff.

The most important factors that hindered park staff in achieving interpretation goals were resource deficits, bureaucratic barriers, and a lack of common goals. First, it is not surprising that all staff resource mentioned deficits as a hindrance to success, since many North American park agencies have reported budget cuts for visitor services (Park People, 2019). Without adequate staff, funding, and political support, it is difficult to achieve goals (Brewer, 2005; Graham et al., 2021). Similarly, resources and management are critical in shaping staff performance and subsequent success in achieve agency goals (Boyne, 2003).

Second, barriers in bureaucracies can hinder empowerment and self-efficacy and the subsequent ability for employees to achieve their desired goals (Clement et al., 2016; Keller & Dansereau, 1995). Within Alberta Parks, planners and managers along with interpretive coordinators and supervisors were especially aware of the negative effects of bureaucracy and obstruction.

Third, respondents report the lack of common goals as a factor hindering interpretive success. While this constraint was most frequently identified by coordinators and supervisors, our study found little evidence of goal misalignment among employee groups. Strategic planning is designed to identify common goals and agree on a course of action to meet those goals (Bryson, 2018), but frontline interpreters use other resources more than park management plans for guidance in their work (Blye et al., 2022). While a lack of common goals and strategic direction was largely discussed by upper-level staff including planners, managers, and supervisors/coordinators of interpreters, the impact is likely felt by frontline staff. This impact may be playing out in their experiences of bureaucracy and lack of innovation. If the agency as a whole was able to articulate direction and clear goals perhaps this would lead to more congruency and efficiency in achieving conservation and visitor service outcomes (Kapos et al., 2008).

### Limitations and Future Research

There are a few limitations of this study. First, we examined a single park agency, which limits generalizability to other agencies. Second, the sample did not include respondents at the executive level. It is possible that executives would have had different priorities for interpretation goals and perceptions about factors that help and hinder interpretation’s goals. However, by working with personnel lists and experienced interpretive managerial staff, we achieved an extensive sample of all other staff directly and indirectly involved in the design and delivery of the park agency.

To increase understanding staff performance and job satisfaction, researchers could expand the comparison of interpretation goals to include the agency’s legislation, policies, and management plans (Boswell, 2006), and to outcomes as reported by visitors (e.g., just after an interpretive experience and some time later) and as desired by visitors (Van der Merwe et al., 2020). Researchers should examine which factors affect the differences in priorities for interpretation goals and resources used (e.g., accountability, metrics, collaboration possibilities). Determining how these priorities translate into interpretive program content, delivery methods, and actual outcomes would help guide future interpretive planning practices. Last, examining priorities for interpretive goals among other park agencies and cultures would enable cross-organizational and cross-cultural comparisons (Yamada et al., 2021), which in turn, would help build theory about goal alignment in a global interpretive context.

### Implications for Practice

This study showed a goal alignment among employee groups, with the goals of positive memories, enjoyment, and connections to place ranking higher than behavior change, attitude change, and learning. Such alignment can help improve agency performance. To continue aligning goals, organizations can promote vertical and horizontal communication, strategic planning, staff engagement, and training and development (Rapert et al., 2002; Torland et al., 2015). At the manager level, alignment increases by clearly communicating goals, building trust with employees, encouraging worker participation in decision-making processes, and providing feedback to staff (Favero et al., 2016). At the frontline staff level, alignment is aided by regular staff debriefings, listing resources to share with interpreters, encouraging interpreters to accompany scientists into the field, co-creating research activities, and seeking input from interpreters about research (Merson et al., 2017).

Despite the focus on goal alignment, sometimes misalignment can helpfully signal the need for change that one group of employees may observe earlier than another group (Franke et al., 2021). For example, frontline interpreters may hear directly from visitors about their interpretation wants and needs, or seasonal interpreters, who have just completed advanced interpretive training, may be exposed to novel approaches to delivering content.

Park practitioners should respond to the factors that helped and hindered park staff in achieving their interpretive goals. Park managers should hire and train supportive supervisors and skilled team members that all levels of staff desire. Park managers should provide training opportunities; in times of budgetary constraint, valuable training can come from more experienced interpreters. Even if park managers cannot overcome resource deficits, they can explain to staff the constraints and the reasons behind budgetary or staffing decisions. Similarly, park managers can support staff in negotiating with their relevant bureaucracy (e.g., faster decisions, timely inputs of “seed” resources to advance an interpretive goal). Last, park managers should promote discussion about goals for interpretation programs as well as for the larger agency.
